# Dr. Hamilton’s Surgical Dispensary

**Published:** 1847-07

**Authors:** Wm. H. White


// BUFFALO MEDICAL JOURNAL
AND
MONTHLY REVIEW.

VOL. 3. JULY, 1847. NO. 2.

### ORIGINAL COMMUNICATIONS.

ART. 1. *Dr. Hamilton's Surgical Dispensary.* Reported by Wm. H. White.

*Encysted Tumour.*—G. D. aged 20. Had a congenital encysted tumour situated at the external angle of the eye; in size and shape it resembled an almond.

Operated by opening directly into the sack, and then removing the sack by evulsion. The second day after operation he again appeared before the class—slight eccymosis, but nearly healed.

*Otorrhoea.*—Mrs. S., Canada; aged 26. Last April contracted a severe cold, which resulted in inflammation of the internal ear; a month after it suppurated, and has continued to discharge until now. This abscess now extends under the integument of the mastoid process. The general health is much impaired.

Dr. H. advises from 15 to 20 drops of Coindet's mixture three times per day; a tea-spoon full of calcined magnesia on going to bed, and the abscess to the syringed out daily with water and castile soap.

*Indirect Inguinal Hernia.*—N. J. T. aged 45. Hernia of both sides of 20 years standing; caused by lifting. The right testis is also diminished in size and flabby. He has worn a truss nearly the whole time (the long continued use has made a depression at both external rings). He is now useing Culver's truss, has used Hull's, but he thinks C. superior.

Recommended to continue same truss.

*Hemiplegia of left side.*—L. C. aged 28. Jan. last, patient had an apoplectic fit, at which time he fell upon the right side of the head; his sight, speech, and power of thought have been impaired ever since. He was cupped and blistered at the time, but did not receive any benefit.

Dr. H. regarded it as a structural disease of the brain and its meninges, but, thought it possible that the internal plate was fractured. Advised laxatives, low diet, and long continued use of seaton in the neck.

*Nebula.*—H. S. D. while crossing the Atlantic a year since, had inflammation of the eyes; which resulted in a nebula of the right eye. The nit. silver has been employed for its removal but without success.

Dr. H. thought this proper treatment, but, as the eye often tires of the same remedy, for a time he would advise sulphate of zinc grs. vi, aqua fluv. ℥i and then if not successful—try again the silver.

*Conjunctivitis.*—Ann M. aged 5. Has a scropulous diathesis; her general appearance denotes debility, appetite poor; inflammation more diffused in the left than in the right eye.

℞. Sulphate of zinc grs. ii; Sulphate of Morphine, grs. ii, distilled water ℥i, applied to the eye three times a day, also a desert spoonful of sulphate of magnesia for four successive nights, as a cathartic.

*Injury of the Muscles and Ligaments about elbow-joint.*—J. F. E. aged 32. Caused by a mill log falling upon the arm four weeks since; within an hour after the accident, Dr. McH. saw it, he pronounced it badly bruised, but not broken. Dr. S., of Lockport also examined it, and agreed with Dr. McH. It is now much swollen, painful when moved, and quite firmly placed in a position one-third flexed.

Dr. H., examined it with care. The bones are not broken, but a contraction of the biceps has taken place; advised to flex and extend it gradually: he might by this in the course of two or three months, regain its use. All liniments and external applications useless.

*Enlarged Lymphatic.*—J. F. aged 15. Has a tumor under the angle of the left infer. maxilla, of two month sstanding; in size it is as large as a pullet's egg. His brothers and sisters have had similar enlargements on differnt parts of the neck which after a few months, disappeared spontaneously.

It is an enlargement of a lymphatic gland. Advised low diet, Coindet's mixture ten to thirty drops three times per day.

*Cicatrization of the integuments and palmar aponeurosis of hand.*

C. D. Caused by a burn 14 years since. During the last few months, it has increased, the finger is now flexed nearly one-third.

Cut the cicatrix and bound the finger back. In about two weeks it was healed and straight.

*Extraction of two teeth from a patient under the influence of the Letheon.*—A soldier appeared before the class with two decayed teeth; the last bicuspid, and first molar. Dr. Snow, Dentist of Buffalo, gave the letheon and extracted the teeth. While its influence was passing off, the patient arose, and with a good deal of enthusiasm exclaimed, "I felt as it I was going to heaven." After it had entirely passed off, he said he did not feel the extraction of the first tooth, the second hurt him some, but not much. He left highly pleased with the operation.

*Labium Leporinum.*—D. S. aged 10. Hare lip, single fissure on the left side, running up nearly to the ala of the nose. It was once operated upon in Ireland, but the stitches tore out.

Dr. H., operated as he usually does, with the scissors and forceps, drawing the parts together with common interrupted sutures, over which he placed adhesive straps. The boy three days after, again presented to the class, at which time, the parts had adhered and the stitches were removed; eight days after the operation, the straps were also removed, and the case was complete and without the slightest prolabium fissure.

*Ptosis.*—J. B. from Toronto. The left eyelid; of long standing; dropped very much.

Dr. H. operated, taking out a leaf-shaped piece of integument, half inch in width in the centre and extending the whole length of the lid, and closing the wound with sutures; it was perfectly successful,

*Amaurosis.*—Mrs. Q., aged 30. The sight began to fail about two years since. She has been under treatment at several periods, but received no benefit. It is a case of congestion of the retina.

Prescribed seaton in the neck, full venesection, light diet, a mild cathartic occasionally.

*Staphyloma.*—A. P. aged 31. Of one year standing; both balls project very much; the vessels highly conjested, the lids granulated, it is the result of acute inflammation.

Dr. H., thought him past recovery but would advise a saturated solution of nitrate of silver, blisters upon the neck, the granulations to be cut off, the bowels to be kept open exercise in the open air, and moderate diet.

*Felon.*—G. R. D. at the second joint of the second finger on the left hand; deep seated.

Opened and ordered bread and milk poultice.

*Staphyloma.*—A son of Peter McK., aged 7 years. The right eye is entirely lost, the left nearly; the child has a scrofulous diathesis. Incurable.

*Enlarged Tonsils extirpated.*—Miss H. D., aged 7 years. They have been increasing for the last four years.

Extirpated with Owen's instrument.

*Indirect inguinal Hernia.*—An old "salt water tar" appeared before the class with a reducable hernia of many years standing and of prodigious size, it was brought on by hard bruises.

Advised to get Chase's Truss.

*Necrosis and Ulcer of the shaft of the Tibia.*—H. P., In 1844 while riding on horse-back, crushed his leg between the horse and a stump, causing a compound fracture of tibia and fibula, it was soon seen by a physician and set, and to keep up extension the straight splint was employed. It did not unite kindly; in the course of two years it resulted in a large ulcer and necrosis; several pieces of bone have been discharged. There is an opening about four inches from the head of the tibia, and one on the same side an inch above the external malleolus, each discharging matter.

Dr. H., examined it but could not discover any loose bone, advised some further delay before submitting to an operation.

*Indolent Ulcer.*—G. F. of ——— two years since was thrown from a wagon and injured the right knee; it immediately caused a swelling which obliged him to keep still for a week; cold water was applied; this reduced it to a small hard bunch situated two inches below the patella. After a few months, it again began to grow and soften. A charcoal poultice was applied, and it suppurated and opened. Ulcer now extended from knee to upper end of the lower third of the tibia; it has been nearly healed over by nitrate of silver in powder; now applying a bark poultice.

Dr. H. said there was no signs of the bone being diseased, and as it was improving he would continue the present treatment.

*Incomplete fistula in Ano.*—J. D., negro, aged 55. Came before the class, his body much flexed, walking with a good deal of pain, complaining of a small bunch near the anus. It proved to be an abscess.

Being freely opened it discharged faceal matter. Ordered to apply a poultice, and in a few days, the radical operation might be made.

*Chronic Conjunctivitis.*—Geo. W. had acute opthalmia resulting in a superficial ulcer upon the cornea of right eye: the system is deranged and weakened. ℞ nit. sil. grs. iii, water ℥i. Tonics.

*Contraction of muscles of the hand.*—Mrs. A. aged 40. Caused by a burn several years since, the tendons are flexed one-third.

Dr. H., said it was a case upon which no prudent surgeon would operate, as these tenotomy operations when applied to the fingers, generally resulted in paralysis of the several tendons, and advised her never to have them cut, but to wear an elastic splint—this would, after a time, bring it straight.

*Scrofula.*—Peter W. aged 34, from Argyleshire, among the highlands of Scotland, left there four weeks since; swelling commenced about two months before he left; his family have never had anything of the kind—neck much disfigured.

Prescribed tinct, iodine 15 gtta. two or three times per day, and to apply it also externally·

*Hydrocele.*—P. B. aged 28. It has existed for many months, the palliative operation has been performed three times. The scrotum having again become much extended and painful, he agreed with

Dr. H., that it would be proper to have the radical operation performed.

Operated, injecting a solution of tinct. of iodine and water—ʒi to ℥i.

*Fracture of Infer. Maxilla.*—C. B., aged 55, from Little Falls—Caused by the kick of a horse fourteen days since; three days after, it was seen and most admirably adjusted by Dr. Green of Little Falls, he useing for support, Cooper's apparatus. One of the molar teeth and a small piece of the jaw was entirely broken out.

Dr. H., dressed it with his own apparatus which possesses several striking advantages over that recommended by any other surgeon.

*Sarcocele.*—J. P., aged 29. Cause not known.

Advised a suspensary bag and a thorough dose of sulphate of magnesia.

*Congenital Talipes.*—An infant daughter of P—— of ——, both feet varus third degree; perhaps the right foot might be considered as fourth degree. The child perfectly healthy.

Operated on both by dividing the tendo Achilles. The left foot could now be brought to the natural situation; the right was improved, but not as much. The tin shoes prepared, were immediately applied; with orders if they were at all painful to loosen them, and to remove them during the few first nights.

*Tumor suddenly formed.*—Son of Rev. L. S. aged 4. Three weeks since, the mother while washing him one morning discovered on the right side of the neck, about an inch above the clavicle on the sterno-cleido-mastoid, a tumor then as large as now, about the size of a hen's egg. Its feel is tense, slightly elastic, not discolored, no pain or tenderness. In its general appearance it resembles an encysted tumor. The day before it appeared, he fell two or three times, once on his stomach, causing him to spit blood. The parents were confident the swelling had never been there before, nor has he been subject to similar swellings. The child has not a very healthy look, no particular evidence of hemorrhagic diathesis. To-day (two weeks after) it is increased in size; tender and slightly discolored. Suppuration is evidently about to take place.

*Abscess of the hand.*—J. S. D. caused by an incision between the thumb and index finger; on the dorsal side of the hand. It is now discharging healthy matter. The edges are elevated and callous.

Opened it, and advised bread and milk poultice.

*Malignant Tumour.*—Mr. F. This patient appeared before the class four weeks since for Dr. H's., opinion. The tumor was situated on the ulnar and palmar side of elbow, over the brachial artery and median nerve, and firmly attached to the bone, and surrounding parts. It was examined very minutely by Dr. H., and he frankly said he could not give a certain diagnosis, but from its situation and its having made its appearance within a year, being without pain and feeling like, and being in the situation of a bursa, he should be inclined to think it such, to be more certain, he opened it with an iris knife; no matter escaped. Having brought it together with adhesive plaster, he requested him to call again in a week. At the end of the time he came, and the cut instead of healing was surrounded by a bright red ring, and a white glassy fluid was exuding from the orifice. It was now examined by several surgeons, and all fully concured with Dr. H., in the opinion that it should be extirpated.

Dr. H., operated May 1st; by his first incision he entered the sack, cutting the cephalic vein, arteria profunda inferior, anastomatica and several muscular branches were also cut and tied; the internal insertion of the biceps was cut. The tumor being very firmly attached, made the operation long and exceedingly difficult. June 16th; tumor is returning; it is malignant.

*Necrosis.*—P. B. R. aged 11 years. The upper extremity of the tibia is necrosed, also enlarged; two openings in the diseased part. ℞. hydriodate of pot. and sarsaparilla.

*Congenital Talipes.*—C. R. B. Black Rock, aged 14 years. Varus 3d degree.

Operated by cutting on the right foot the tendo achilles, on the left foot the tendo achilles and the tibialis auticus: immediately after the operation, the feet could be forced out to the 2d degree. Will apply Stromeyer's apparatus this afternoon.

*Ectropium.*—Miss Mary A. F. The lower lid of left eye inverted one-fourth of an inch, caused by an abscess following small pox. Operated by taking out a triangular piece and bringing the parts together by sutures.

